# On the way to 40 years: the new CSP website

**DOI:** 10.1590/0102-311XEN174623

**Published:** 2023-10-13

**Authors:** Luciana Correia Alves, Marilia Sá Carvalho, Luciana Dias de Lima

**Affiliations:** 1 Instituto de Filosofia e Ciências Humanas, Universidade Estadual de Campinas, Campinas, Brasil.; 2 Programa de Computação Científica, Fundação Oswaldo Cruz, Rio de Janeiro, Brasil.; 3 Escola Nacional de Saúde Pública Sergio Arouca, Fundação Oswaldo Cruz, Rio de Janeiro, Brasil.

Since 2021, CSP has developed a series of activities and investments to improve its structure and internal processes ^1^. An academic journal of the size of CSP has not only the role of gathering and validating quality scientific research, but also being a reference for non-specialists who seek a safe and reliable source of information on issues of interest to public health and society. In 2024, CSP will celebrate its 40th anniversary and some new features are being prepared.

As the first initiative of the celebrations, in July of this year, we launched the new CSP website, with several changes and new functionalities, responding to aspects related to indexing requirements and relations with authors and readers. The design is more appealing and the navigability has become dynamic and interactive.

Scientific journal websites play an important role, since they allow research results to be accessed by anyone worldwide. Since 1998, when CSP joined the SciELO network, its entire collection has been open access and free of charge for authors. Continuous publication was adopted in 2016, when the journal became online-only. Even with the agility provided by digital publication, the organization in monthly issues was maintained, differentiating regular numbers from the thematic ones and thus facilitating the search.

An aspect to be highlighted is the responsiveness of the new website. It can now adapt its layout to all devices, from desktop computers to tablets and smartphones. This is one of the most significant novelties of this new design: content can be read anywhere and by different readers profilers.

The second important change was on the presentation page of each article. The format facilitates reading and makes the bibliographic references and illustrations referring to each excerpt of the manuscript accessible by a simple click. Citation indicators and metrics of the articles, the so-called altmetrics, were also incorporated both into the usual indexers and into the data of accesses in different media.

Many innovations of internal function of CSP are of interest to authors and consultants. Our entire collection can now be accessed not only through SciELO, but also on the servers of the Sergio Arouca National School of Public Health, Oswaldo Cruz Foundation (ENSP/Fiocruz). We will also soon shut down the Article Evaluation and Management System (Sistema de Avaliação e Gerenciamento de Artigos - SAGAS). The electronic publication process will be linked to the Open Journal System (OJS), which we hope to put on air as soon as the adaptations we deem necessary are completed.

With the new website, we intend to expand the space for discussion and encourage greater engagement of the scientific community and society as a whole. We seek to increase our presence on social networks, produce videos, stimulate activities aimed at scientific dissemination and, of course, present all this in an integrated and accessible way for those who read our work. We have maintained our tradition of selecting images for each issue. Each year we choose a thought-provoking and enchanting theme: street art, historical buildings of science, popular demonstrations, among others. A website is not just a scientific article. It is a way of bringing qualified scientific information to the public with beauty and art ^2^.
